# Supplementary material for: Gene loss and relaxed selection of plaat1 in vertebrates adapted to low-light environments
Source: bioRxiv. 2023 Dec 13:2023.12.12.571336. Preprint. [Version 1] doi: 10.1101/2023.12.12.571336 (PMC10760033; doi:10.1101/2023.12.12.571336)
Supplement: Supplement 4 — Supplementary Figure 1. Bar plot depicting the number of deletions or premature stop codons (PSCs) in each region of plaat1 for (A) sighted versus low visual acuity and blind (LVAB) mammals, and (B) sighted versus LVAB fishes. Genes are counted only once if they have one or more PSC or deletion to reduce bias caused by degraded genes having multiple deletions/disruptions. Numbers under bars indicate total number of sequences (species) that fell into that category. P-values derived from Fisher’s exact tests. Supplementary Figure 2. Gene tree of plaat3 for all mammals included in selection analyses, generated using aBSREL Full adaptive model (Smith et al. 2015). Supplementary Figure 3. Topology of all sauropsids used in analyses next to the protein alignment of plaat1 for each species. Image generated in iTOL. [file NIHPP2023.12.12.571336v1-supplement-4.pdf]

bioRxiv preprint doi: https://doi.org/10.1101/2023.12.12.571336; this version posted December 13, 2023. The copyright holder for this preprint (which was not certified by peer review) is the author/funder, who has granted bioRxiv a license to display the preprint in perpetuity. It is made available under aCC-BY 4.0 International license.

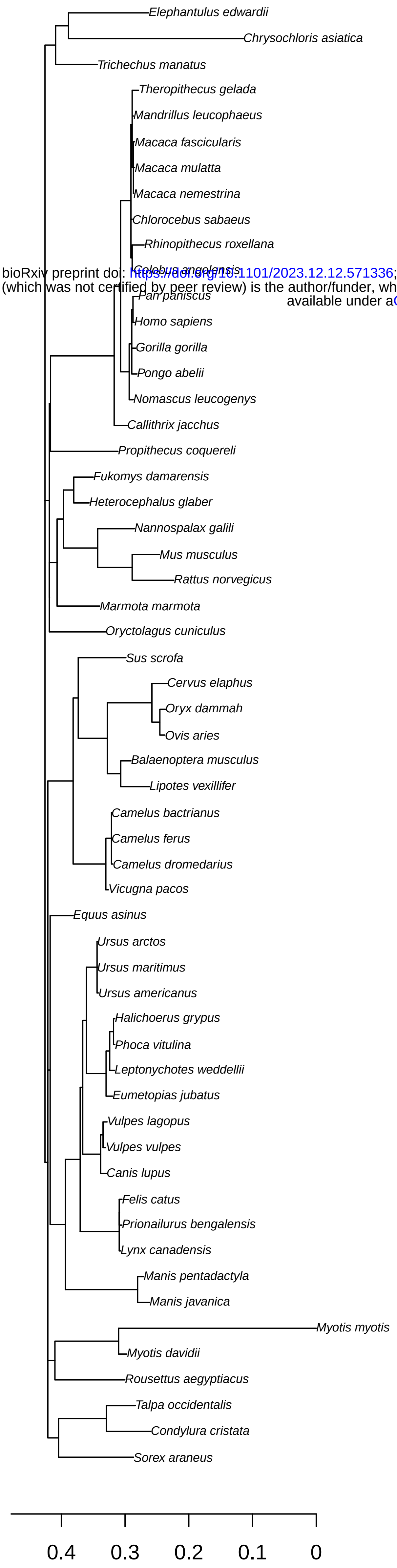

bioRxiv preprint doi: https://doi.org/10.1101/2023.12.12.571336; this version posted December 13, 2023. The copyright holder for this preprint (which was not certified by peer review) is the author/funder, who has granted bioRxiv a license to display the preprint in perpetuity. It is made available under aCC-BY 4.0 International license.

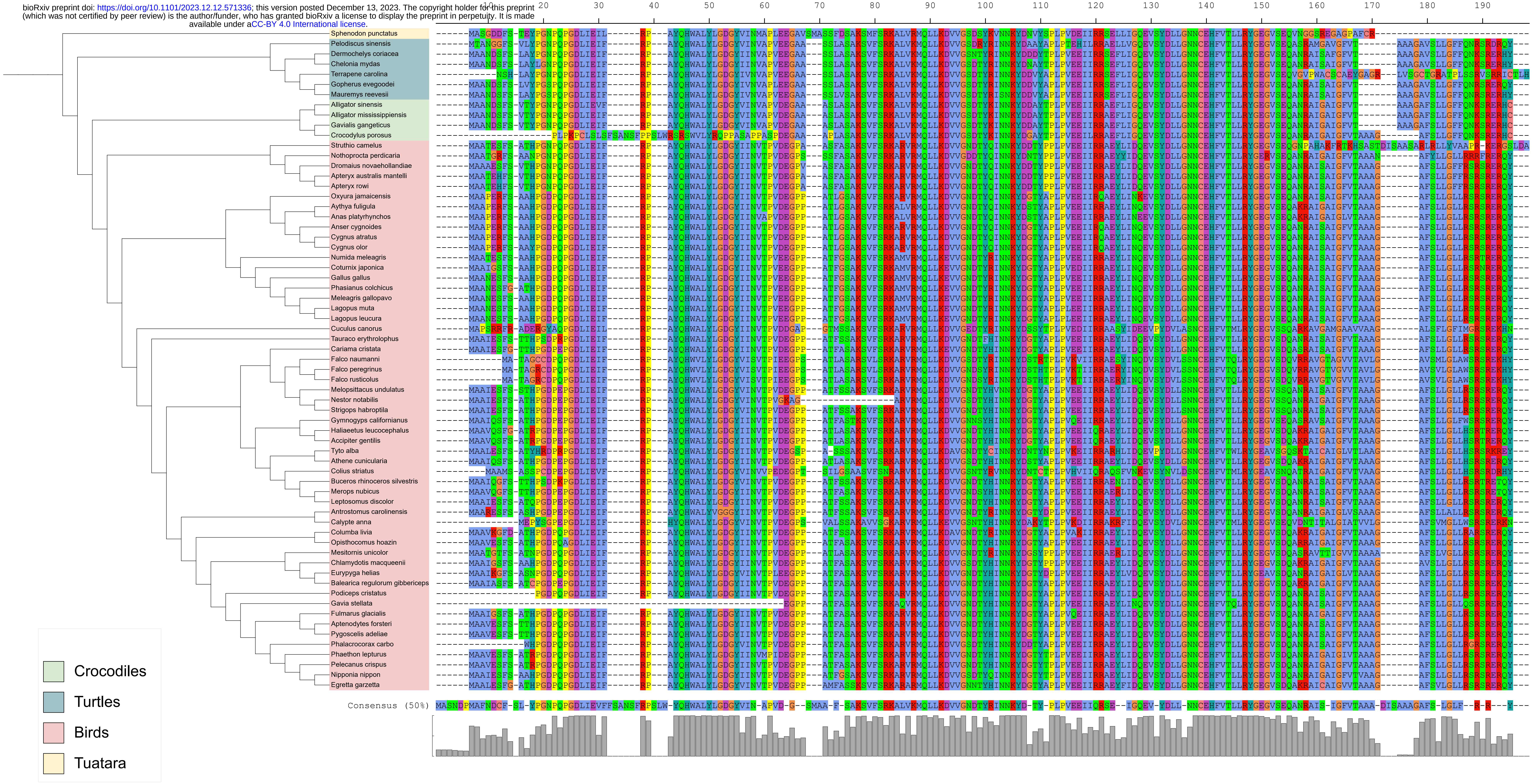

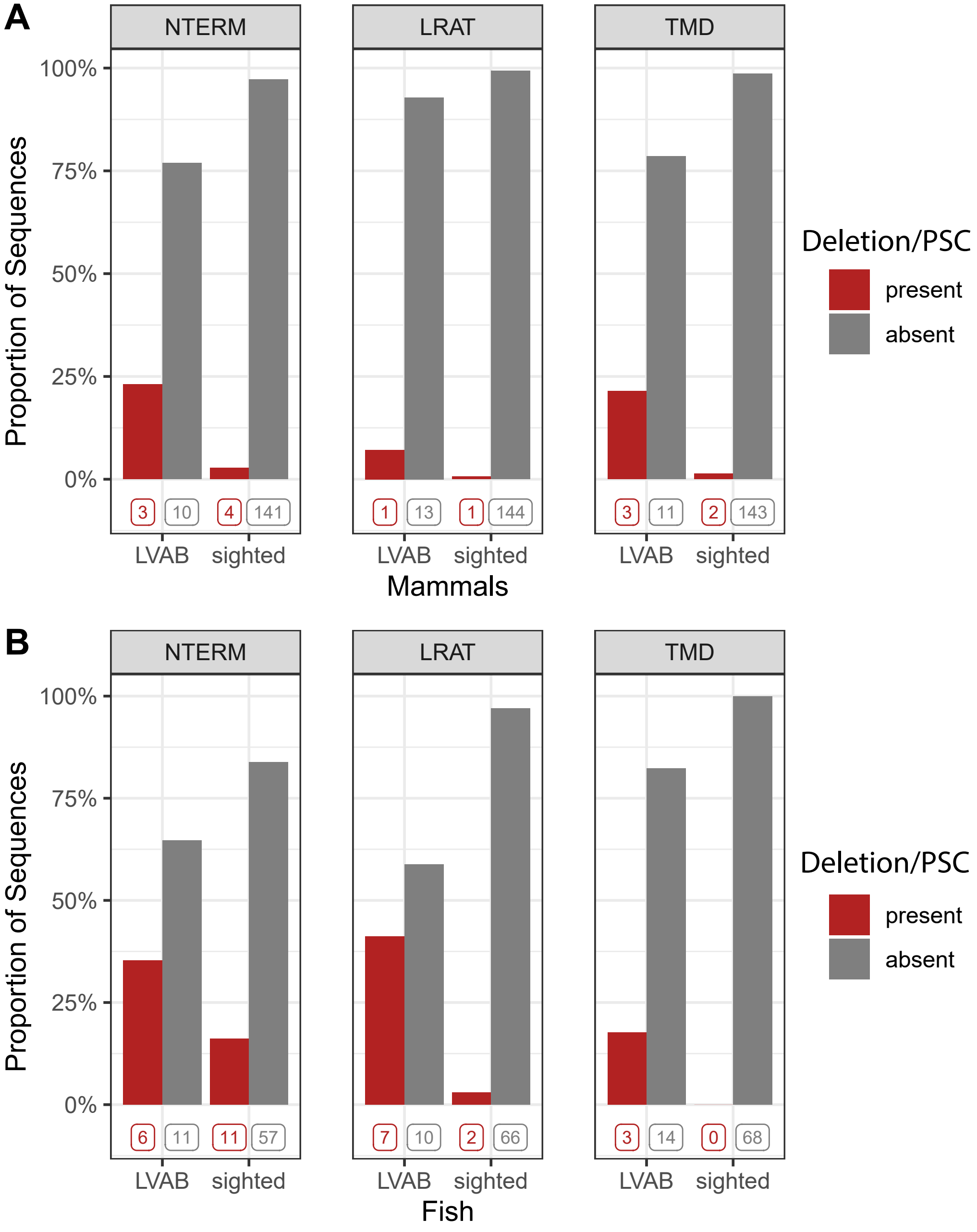
A
NTERM
LRAT
TMD
Proportion of Sequences
100%
75%
50%
25%
0%
3
10
4
141
1
13
1
144
3
11
2
143
LVAB
sighted
Mammals
Deletion/PSC
present
absent
B
NTERM
LRAT
TMD
6
11
11
57
7
10
2
66
3
14
0
68
Fish
